# Natural Variability in Parent-Child Puzzle Play at Home

**DOI:** 10.3389/fpsyg.2021.733895

**Published:** 2021-09-16

**Authors:** Nicole Pochinki, Dakota Reis, Marianella Casasola, Lisa M. Oakes, Vanessa LoBue

**Affiliations:** ^1^Department of Psychology, Rutgers University, Newark, NJ, United States; ^2^Department of Psychology, Cornell University, Ithaca, NY, United States; ^3^Department of Psychology, University of California, Davis, Davis, CA, United States

**Keywords:** puzzles, spatial language, spatial skill, play, parent-child interactions

## Abstract

Here, we observed 3- to 4-year-old children (*N*=31) and their parents playing with puzzles at home during a zoom session to provide insight into the variability of the kinds of puzzles children have in their home, and the variability in how children and their parents play with spatial toys. We observed a large amount of variability in both children and parents’ behaviors, and in the puzzles they selected. Further, we found relations between parents’ and children’s behaviors. For example, parents provided more scaffolding behaviors for younger children and parents’ persistence-focused language was related to more child attempts after failure. Altogether, the present work shows how using methods of observing children at a distance, we can gain insight into the environment in which they are developing. The results are discussed in terms of how variability in spatial toys and spatial play during naturalistic interactions can help us contextualize the conclusions we draw from lab-based studies.

## Introduction

Spatial skills are central for everyday functioning, allowing us to encode the features, locations, and orientations of objects, as well as mentally manipulate this information. Spatial skills not only make it possible to interpret maps and diagrams, but also they are important predictors of later achievement across diverse STEM disciplines (Wai et al., 2009; Uttal and Cohen, 2012). For decades, research has documented a significant and robust relationship between spatial skills and mathematics performance over the course of development (Smith, 1964; Guay and McDaniel, 1977; Brown and Wheatley, 1989; Casey et al., 1995; Shea et al., 2001; Wai et al., 2009; Pyers et al., 2010; Cheng and Mix, 2014; Verdine et al., 2016, 2017). As a result, identifying factors that might influence the development of spatial skills in early childhood has received a great deal of attention in the literature.

For example, researchers have examined children’s constructive play, or play with toys that involve the manipulation of objects in space, such as jigsaw puzzles, shapes, or construction blocks. A large body of research has reported a positive relationship between constructive play in childhood and both advanced concurrent spatial abilities (Connor and Serbin, 1977; Serbin and Connor, 1979; Caldera et al., 1999) and enhanced spatial skills later in development (Newcombe et al., 1983; Baenninger and Newcombe, 1989; Dearing et al., 2012; Levine et al., 2012; Nazareth et al., 2013; Jirout and Newcombe, 2015). Further, a handful of interventions studies have shown a causal relation between children experiences with constructive play, and a subsequent increase in various spatial skills (Casey et al., 2008; Bower et al., 2020; Schröder et al., 2020).

Importantly, such constructive play often occurs during interactions with parents. Thus, parents’ behavior during such play may be important for developing spatial abilities as well. For example, Levine et al. (2012) found that parents used more spatial language, including words describing the spatial properties of objects (e.g., “big,” “little,” “flat,” and “edge”) when their children were engaged with more challenging puzzles. This finding is important because children who hear more spatial language perform better on spatial tasks (e.g., Szechter and Liben, 2004; Dessalegn and Landau, 2008; Casasola et al., 2009, 2020). Thus, exposure to language is one possible mechanism for how play with parents shapes children’s developing spatial abilities.

Parents may also support children’s emerging spatial skills during constructive play by giving feedback, structuring the task, and modeling ways to problem solve during constructive play (Wood et al., 1976; Gauvain et al., 2002; Mulvaney et al., 2006; Ralph et al., 2020; Thomson et al., 2020). Children whose mothers provided more support or scaffolding during a spatial task performed better on a cognitive capability test that included measures of spatial ability (Mulvaney et al., 2006). Further, several studies have shown parents who better communicate task objectives and provide appropriate feedback have children who perform better on spatial tasks and tests of spatial concepts (Casey et al., 2014; Lombardi et al., 2017). Thus, scaffolding is another mechanism by which parents may influence children’s spatial development during play.

Altogether, a large and growing literature suggests that several factors—including constructive play, exposure to spatial language, and parent scaffolding—may all play a role in shaping the development of children’s spatial skills. Importantly, many of these studies have been conducted outside of the home, typically in a lab setting, with specific constructive play toys and tasks provided to parents and children. Although such experimental control allows us to derive conclusions based on standard conditions, the sole use of such assessments is limited, as children’s behavior, along with parents’ behavior with their children, might differ in the lab when compared to this behavior at home. Moreover, the constructive toys provided for a study in the lab may differ from those with which children typically play. Indeed, parents themselves have a great deal of control over what types of spatial toys they make available for their children, and they have many options to choose from. A simple google search for children’s spatial toys produced over 5 million results, which can be narrowed down by the type of spatial toy in which a parent is interested, along with price, and the age and gender of their child. And there is evidence that the types of toys with which children play might bring about specific types of behaviors. In fact, researchers have even suggested that gender differences in spatial abilities might be attributable to differences in the toys parents select for girls versus boys (Todd et al., 2016; Coyle and Liben, 2020).

The COVID-19 pandemic has put a number of constraints on researchers’ ability to collect data with children in the lab and in some ways, necessitated new approaches to study development. Here, we show how we used a videoconference platform (Zoom) to study spatial play at home from a distance, along with the spatial and constructive toys that parents typically choose for their children. The existing studies that have examined children and their parents playing with toys in the home have focused on the relationship between the *frequency* of spatial play and parent support (Levine et al., 2012), or parent language and children’s performance on spatial tasks (Mulvaney et al., 2006; Pruden et al., 2011; Polinksy et al., 2017; Ralph et al., 2020). Here, we asked a different question. Specifically, we sought to characterize the variability in various factors linked to spatial skills in children during their naturalistic play with the spatial toys they had at home. We explored variability in the types of puzzles families of 3- and 4-year-old children interact with in their homes, and the nature of those parent-child interactions during naturalistic play. We conducted the study over Zoom, and simply recorded parents and children as they played.

## Materials and Methods

### Participants

Children between the ages of 3 and 4years and their parents were recruited *via* a Rutgers University maintained database to participate in an online study investigating the development of spatial skills in children ages 3 and 4years. Forty-two dyads participated in the study. Eleven were not included in our final sample due to either deviation from the protocol (*N*=3) or lack of puzzles at home (*N*=8). The final sample included 31 children (14 female, *M*_age_=44.6months, *SD*=6.32, *Range*=35.8–55.3months) and their parents. All except for two parents presented as female. Families identified as White (*N*=28), Asian (*N*=2), or Mixed Race (*N*=1). Across all racial categories, four identified as Hispanic or Latino (three were White and one was Mixed Race). All caregivers had earned a bachelor’s degree and 23 held advanced degrees. Our sample was middle class, with 22 families reporting an annual income above $100,000, and only one family reporting an annual income below $40,000. The Rutgers Institutional Review Board approved all procedures.

### Procedure

Parents were invited to participate in an online study. Once an appointment was scheduled, families were emailed a link to a secure online survey *via* Qualtrics. This survey contained a consent form and an extensive questionnaire designed to describe the children’s home playing environment. This questionnaire was part of a larger study designed to quantify the number and kinds of spatial toys in the participants’ homes, and most of it will not be reported here. In one section of the survey, parents were presented with sample photographs of jigsaw puzzles and puzzle boards and were asked if they had those or similar toys at home. Parents were then asked to submit photos of those toys. The photos were used to code properties of the puzzles parents and children played with during our study.

**Figure 1:**
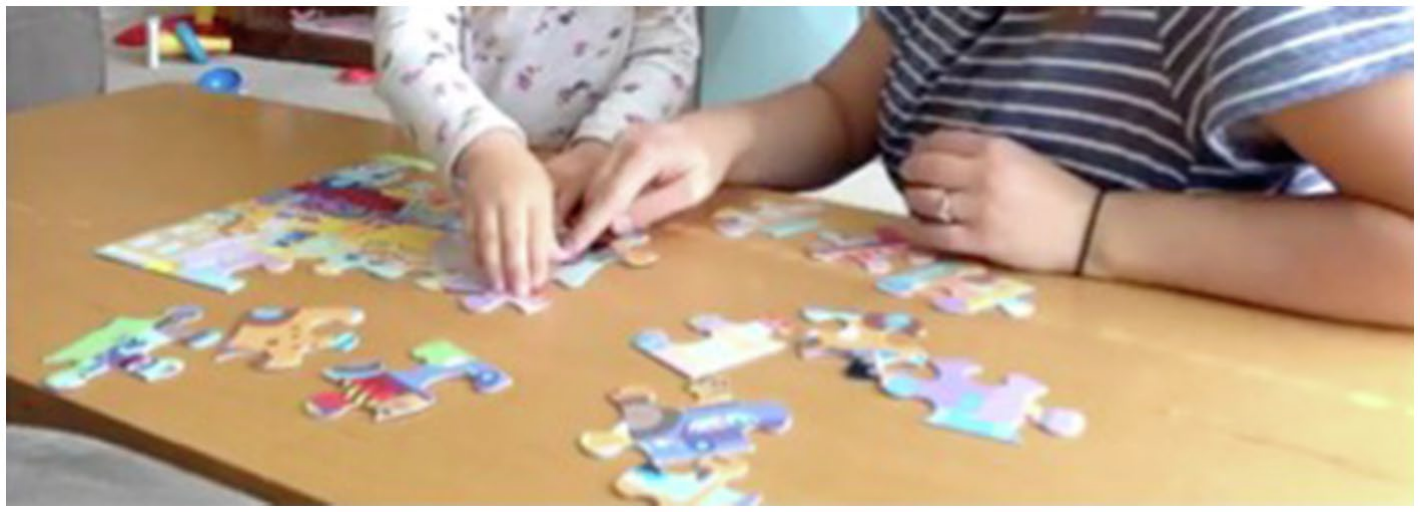
Camera set-up for the puzzle session.

One day prior to the study, participants received a reminder email informing them that they would be playing with puzzles. Parents were asked to select two puzzles from the ones they described in the survey for use during the study. The study itself was conducted on Zoom. On the day of the study, a researcher informed participants that they would be recorded playing with their child. Parents were asked to set up the camera in a high angle so all the pieces and playing space were in view and the researcher was able to look down at the participant’s hands and all the pieces (see Figure 1). The researcher asked the parents to retrieve the previously selected puzzle(s). Parents and children were then instructed to play with each puzzle as they normally would for 10min. If participants finished both puzzles before the 10-min mark, they were asked to retrieve additional puzzles. Thus, some children completed one puzzle during the 10-min session, while others completed up to 5. If they did not complete the puzzle during the 10-min session parents and children were given the option to finish. The researcher turned off her camera during the play period so that the parent and child could no longer see the researcher observing, and the researcher did not interrupt the play period before the 10-min mark.

### Coding

Coders watched the recorded play session to categorize the puzzles’ difficulty and to identify instances of specific child and parent behaviors. Children’s insertion attempts, parental scaffolding behavior, and parental language were all coded using the open-source behavioral coding software, Datavyu.^1^

#### Puzzle Difficulty

**Figure 2:**
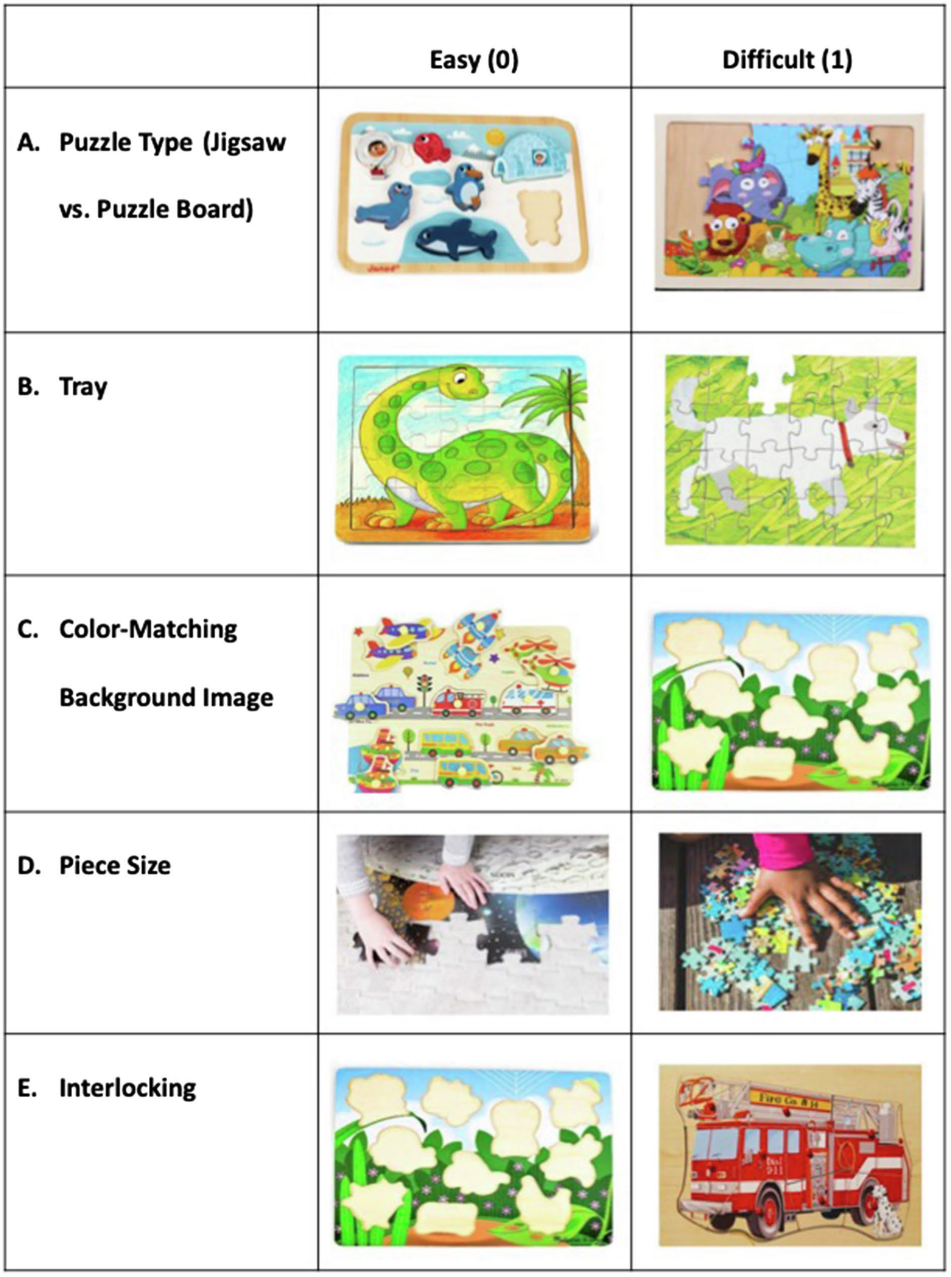
Puzzle dimensions that were coded for difficulty. **(A)** Puzzle type, **(B)** Tray, **(C)** Color-matching background image, **(D)** Piece size, **(E)** Interlocking.

Parents chose puzzles that varied on a number of characteristics. One coder viewed all sessions and characterized all of the selected puzzles based on dimensions that might influence puzzle difficulty. There were five nested dimensions, each that were assigned a value of 0 (easiest) to 1 (most difficult). The first dimension was Puzzle type, which referred to whether the puzzle was a board puzzle (0) or jigsaw puzzle (1) (see Figure 2A). Puzzles were further coded for whether or not they had a tray (0 if they did and 1 if they did not; Figure 2B). Puzzles that had a tray were then coded for whether they contained a background image that matched the puzzle piece (0) or no background image (1) (see Figure 2C). Puzzles that contained large pieces (i.e., pieces that were larger than the child’s hands) were considered easier (0) than standard jigsaw puzzles (1) (see Figure 2D). Finally, puzzles were coded for whether or not they involved interlocking pieces (no interlocking=0 and interlocking=1; see Figure 2E). These dimensions were summed. For example, a jigsaw puzzle (1) with a tray (0) that contained a background image (0) with large (0) interlocking (1) pieces would receive a score of 2.

The number of pieces in each puzzle was also coded from the videos of the play session and from the puzzle photos submitted through the Qualtrics questionnaire. If information about the number of pieces was missing, an online search was conducted to identify the puzzle and obtain the specifications from the manufacturer’s Web site. A second coder coded 25 puzzles out of a total of 65, and reliability was calculated for all the classifications described above (*κ*=1) and for the number of pieces (percent agreement=96%).

A puzzle difficulty composite score then was created by adding the binary values of all the coded difficulty dimensions and a code ranging from 1 to 5 based on the number of pieces such that the puzzles contained (i.e.,1 to 10 pieces received a score of 1; 11 to 20 pieces received a score of 2; 21 to 30 pieces received a score of 3; 30 to 40 pieces received a score of 4; and greater than 40 pieces received a score of 5). The final puzzle difficulty score ranged from 1 to 10, where a score of 10 was the most difficult.

#### Parent Behaviors

Two coders identified parent scaffolding events in the play session. Scaffolding events consisted of the sum of four different behaviors: (1) removing a piece that was placed in an incorrect space by the child, (2) helping by handing the child individual pieces or rotating pieces for the child, (3) pointing or outlining to a piece or a space in the puzzle, (4) pointing or outlining to the pictorial representation of the puzzle. Inter-rater reliability was calculated for piece removal (*κ*=0.85), helping (*κ*=0.82), pointing to (*κ*=0.81) or outlining (*κ*=0.74), a piece or space and pointing to/outlining a pictorial representation (*κ*=0.92). We created a total scaffolding score by summing the instances of each of these behaviors. In addition to scaffolding, we also coded instances where parents inserted a piece into the puzzle for the child (*κ*=0.87). This final code was not included in the total scaffolding behavior score.

#### Parental Language

One coder transcribed all parents’ utterances. We defined utterances as vocalizations that were separated by grammatical closure, intonation contour, or prolonged pausing of more than 2s. Three raters then coded each utterance to assess whether it contained spatial language (percent agreement=95%), praise (percent agreement=95%), or persistence-focused language (percent agreement=99%). Areas of disagreement were noted and resolved *via* discussion, ultimately resulting in consensus.

Spatial language was coded using a coding scheme developed by Cannon et al. (2007). Spatial language included any mention of spatial dimensions, shapes, locations and directions, orientations and transformations, spatial features, and properties. Examples of utterances coded as containing spatial language are “where’s the flat edge?”, “but I think you might need to rotate it a little,” and “this is a big puzzle.” Utterances that contained more than one spatial word were not differentiated from those that contained only one spatial word. We only included spatial terms that were in reference to the construction of the puzzles and omitted terms that were unrelated to the puzzle (i.e., “Your blanket is under the bed”), or unrelated to its construction (i.e., “Put it in/on the puzzle”).

In addition to spatial language, which has been associated with children’s spatial ability in previous research, we also coded praise and persistence-focused language, which have been linked to more general engagement and persistence in children (Kelley et al., 2000). Praise was coded using a coding scheme developed by Gunderson et al. (2013) and included utterances that positively evaluated the child or the child’s actions (e.g., “You’re good at puzzles”; “good job”), or utterances that expressed general positive valence toward the child but not directed at any specific action (e.g., “Awesome!”; “Yay!”). Persistence-focused language was coded using a coding scheme developed by Lucca et al. (2019) and consisted of utterances that were focused on trying or repeated attempts to complete a goal-directed action. Frequently, this consisted of phrases that explicitly referred to acts of trying (e.g., “You’re trying so hard!”).

#### Child Behaviors

First, a trained coder watched the play sessions and identified children’s insertion attempts. An insertion attempt was defined as the first time the child took one puzzle piece and proceeded to either join it with one or more additional pieces or place it in an opening in a puzzle tray. An insertion attempt could be either *successful* if the child placed the piece in the correct space or *unsuccessful* if the child failed to insert the piece correctly and proceeded to place the piece back down on the floor or table. Each time the child attempted to insert the same piece in any opening or location was counted as a single event, which ended when the child either successfully inserted the piece or placed it down. A second researcher coded 25% of the participants and reliability was calculated for the event matching by both coders; reliability was calculated for both correct (*κ*=0.88) and incorrect insertions (*κ*=0.76).

After coding initial insertion attempts, a trained coder went back to each insertion attempt and counted the number of times the children unsuccessfully attempted to insert a single piece before either successfully inserting it or putting it down. An unsuccessful attempt was coded every time the child tried to insert the piece into a different place in the puzzle or in the same place but in a different orientation. A different orientation was defined as a rotation of the piece more than 90 degrees. A second researcher coded 25% of the insertion instances for each participant. Reliability was calculated for the number of insertion attempts (*κ*=0.81).

## Results

### Data Analysis Plan

The main goals of this study were to describe the range of puzzles families selected for the play session, to examine parents’ naturalistic behavior with their children at home while playing with each puzzle, and to examine the relation between parent’s scaffolding and spatial language and children’s behavior with the puzzles. Upon initial visualization of the data, we observed a great deal of variability in all of the variables we measured. Thus, instead of running a large number of inferential statistics, we primarily provide descriptive data of both parents’ and children’s behaviors with the puzzles that they chose to interact with at home. Then, we normalized our measures by totaling the number of behaviors in each 1-minute interval, and then averaging across those intervals, and ran a correlation matrix on puzzle difficulty level, parenting variables (e.g., parent scaffolding, number of parental insertion attempts, parental spatial language, parental persistence-focused language, and parental praise), and child variables (e.g., age, children successful attempts, children overall attempts, and attempts after failure). Finally, we ran a set of simple gender comparisons across all of normalized data, given that gender differences in spatial abilities have been reported in previous research (Levine et al., 2005, 2016; Pruden et al., 2011).

### Puzzle Difficulty

**Figure 3:**
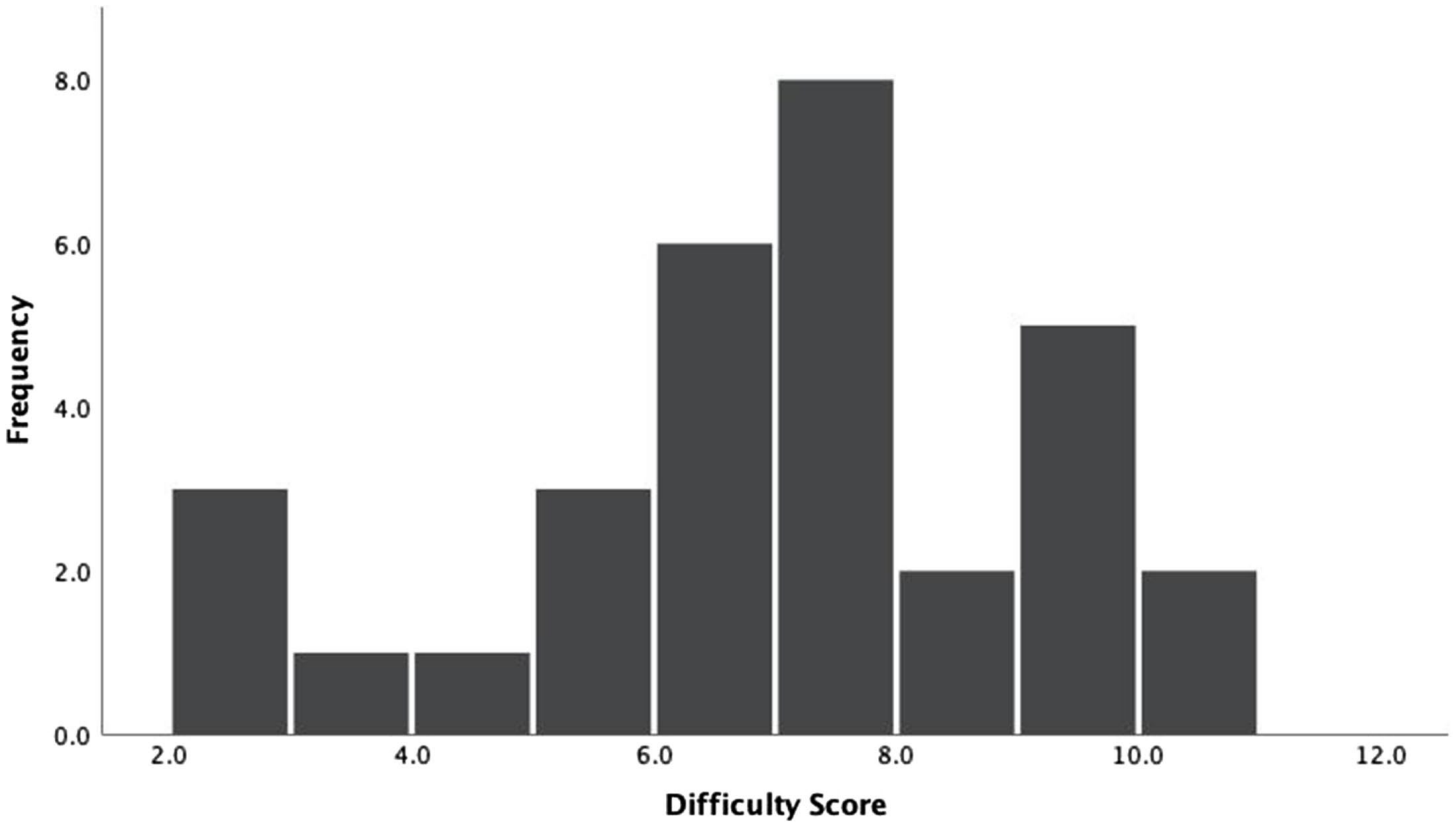
Distribution of difficulty scores across puzzles.

As mentioned above, the puzzles that participants typically played with in their homes varied widely, which is evident by the distribution of difficulty scores across puzzles (see Figure 3). The mean puzzle difficulty score was 6.56 (*SD*=2.17), and the difficulty scores spanned nearly the entire coded range from 2 to 10. Only five participants played with puzzles with a relatively low difficulty score that ranged between 2 and 4; the majority of participants played with puzzles that had a difficulty score in the middle of the range (*N*=17, between 5 and 7), and nine additional participants played with puzzles that were more difficult, ranging in score from 8 to 10.

### Parent Language and Behaviors

**Figure 4:**
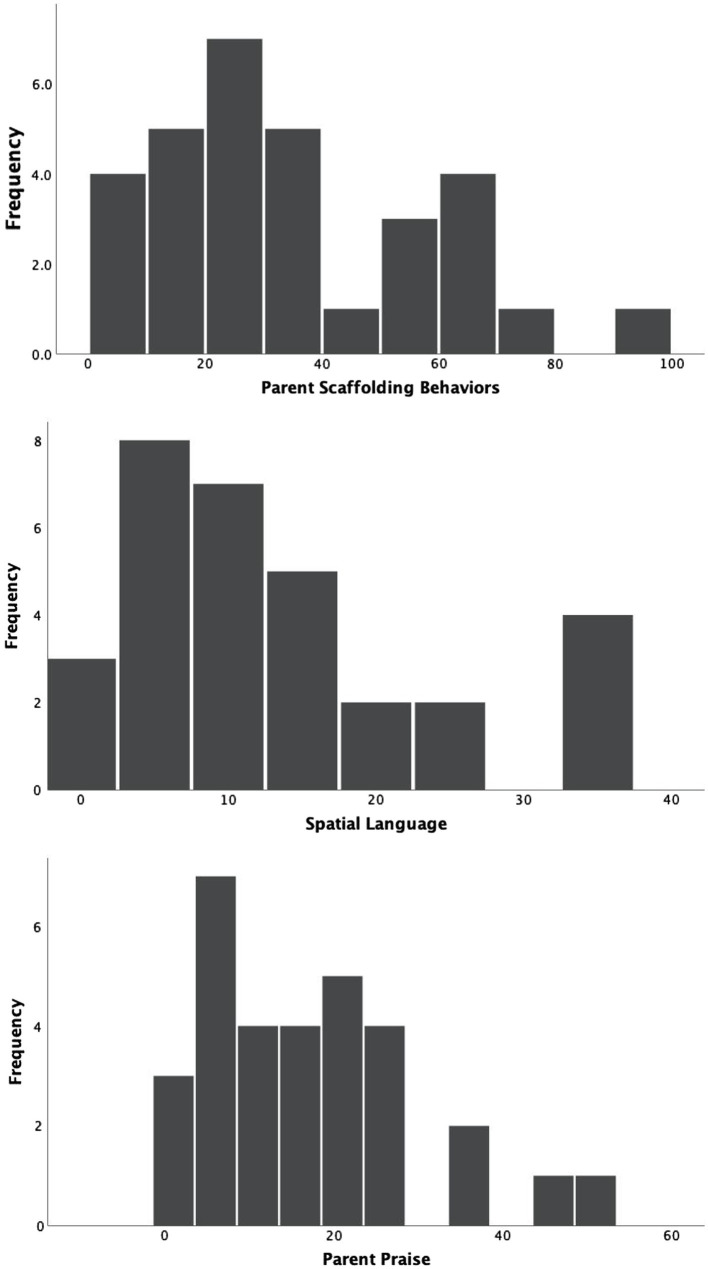
Distribution of parent behaviors.

The distribution of parents’ scaffolding, use of spatial language, and praise is in Figure 4. Two things are immediately clear. First, parents were highly variable, with some parents exhibiting high levels of these behaviors and other parents exhibiting low levels of these behaviors. It is possible that some of the variability in the number of behaviors may be due to variation in the length of the session. Although parents and children were encouraged to play for 10min, some dyads played for less and others played for longer (*M*=9.77min, *SD*=1.6min, range 5.6min–12.1min). To examine whether the length of the session was related to the frequency of parent or child behaviors, we conducted a series of correlations. None of the relations between the duration of the session (in seconds) and parent or child behaviors were statistically significant (*p*’s>0.05). However, we normalized the data for all inferential statistics (see Section “Data Analysis Plan”).

Second, the distributions for the parent behaviors are very similar, with relatively low levels of the behaviors occurring more frequently than relatively high levels of the behaviors. Further, there is some evidence that the same parents were exhibiting relatively high or relatively low levels of some combinations of these variables. For example, parent use of praise per minute was related to parent spatial language per minute, *r*(31)=0.52, *p*<0.05, and the relation between parent praise and parent use of persistence-focused language per minute was approaching significance, *r*(31)=0.33, *p*=0.07. This result suggests that there are effects of parental talk in general. Further, there were small, non-significant correlations between parent scaffolding behaviors and spatial language events per minute, *r*(31)=0.28, *p*=0.13, and praise, *r*(31)=0.26, *p*=0.17, suggesting that there were also parental behaviors specific to child behavior in this task.

Interestingly, utterances containing persistence-focused language were relatively rare, *M*=1.61 (*SD*=1.61), ranging from 0 to 5 across the session as a whole. Fifteen parents did not produce any utterances with this type of language at all.

To further understand parents’ scaffolding behaviors, we examined separately the individual behaviors we coded. Recall that we coded parents’ removal of an incorrectly placed piece, handing or rotating pieces, pointing or outlining puzzle space, and pointing or outlining pictorial representations of the puzzle. Parents more often pointed to or outlined the pieces or the puzzle (*M*=24.29, *SD*=18.07), than rotated or handed their child a puzzle piece (*M*=9.97, *SD*=11.94). Some parents simply inserted pieces into the correct places in the puzzle for the child, *M*=5.06 times per child (*SD*=7.33). There were large individual differences in this behavior; 21 parents rarely, if ever, inserted a piece for their child (ranging from 0 to 3 pieces), whereas 10 parents inserted between 7 and 30 pieces for their children.

### Child Behaviors

**Figure 5:**
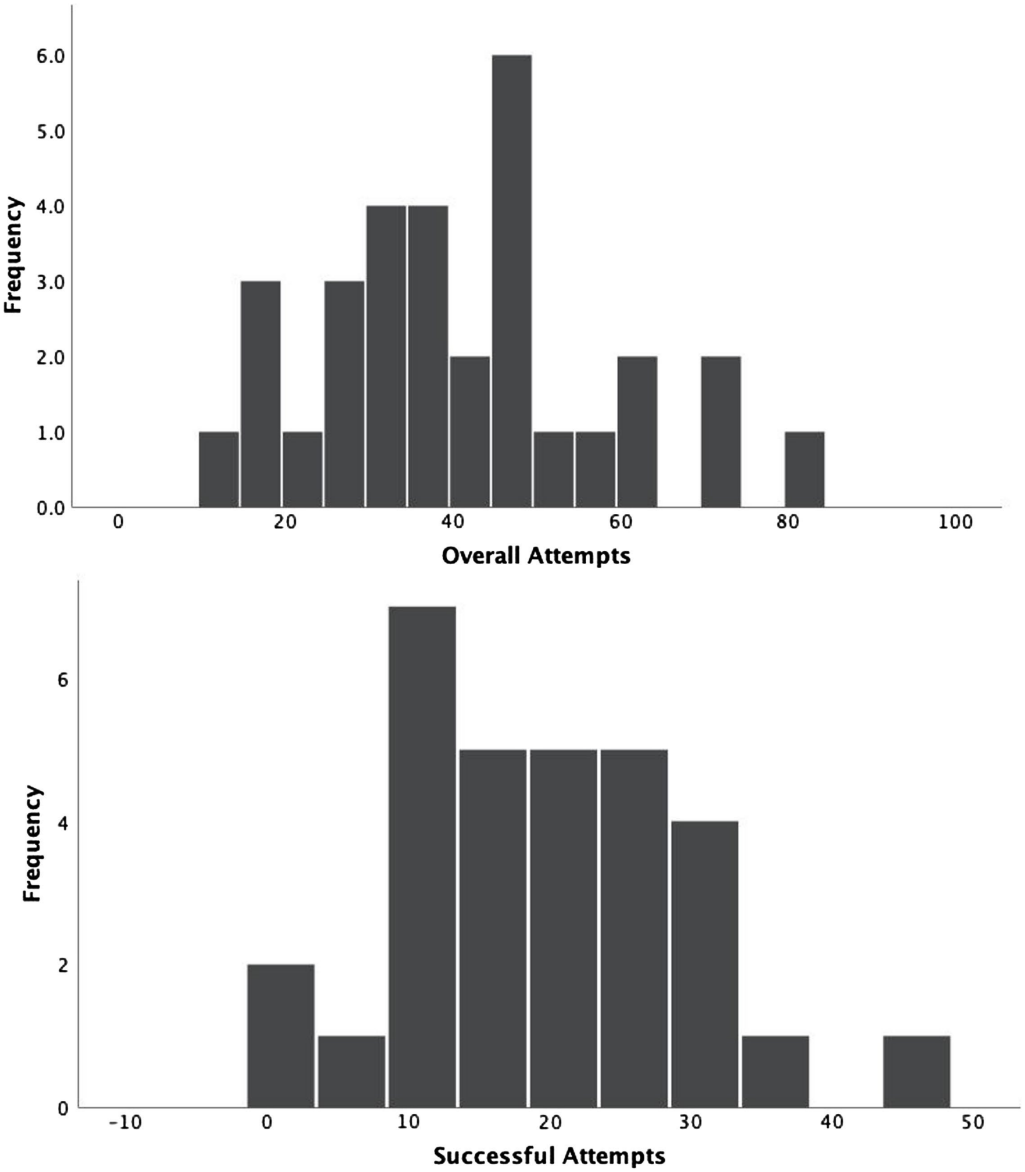
Distribution of children’s attempts.

Children’s behaviors were also extremely variable. The distribution of total attempts and successful attempts to insert a piece is in Figure 5. In terms of attempts, children ranged from making as few as 12 attempts to making as many as 81 attempts, suggesting individual differences in how interested children were in the puzzle. Children’s successful insertions ranged from 1 to 41. The proportion of successful attempts ranged from 3 to 86%, again showing the extreme variability in children’s behaviors.

We also coded how many times children attempted an insertion following a failed attempt. On average, children made 14.61 (*SD*=9.5), such attempts ranging from 2 to 37 attempts. Out of the 250 events where children tried to reinsert a piece upon failure, 66% had a successful outcome eventually.

### Relations Among Variables

Next, we examined how parental behaviors were related to child behaviors during play. To account for the fact that participants’ play time varied (*M*=9.77min, *SD*=1.6, range 5.6min–12.1min), we normalized our measures by totaling the number of behaviors in each 1-minute interval, and then averaging across those intervals. Thus, our measures for these analyses were the number of behaviors or utterances per minute.

First, we examined how our measures were related to child age. The only relation between parental behaviors and child age was a negative correlation between age and parent scaffolding, *r*(31)=−0.38, *p*<0.01. Parents provided more scaffolding behaviors for younger children. It is also noteworthy that there was a small, non-significant relationship between age and children’s successful attempts, *r*(31)=0.28, *p*=0.12, with older children demonstrating more successful attempts than younger children.

Interestingly, despite the wide variation in puzzle difficulty, we found that few child or adult behaviors were related to puzzle difficulty. There was no clear relation to child age, to parental scaffolding or language. The relation between puzzle difficulty and children’s successful number of insertion events was approaching significance, *r*(31)=−0.32, *p*=0.08. Not surprisingly, children were less likely to successfully insert a piece in more difficult puzzles. Note that we conducted a second set of correlations after removing the number of pieces from the difficulty score, as the number of pieces might have skewed the results. However, the results were the same.

We also found that parent and child’s behaviors were related. In particular, the number of children’s insertion attempts after failure was positively related to parents’ persistence-focused language, *r*(31)=0.46, *p*<0.01, suggesting that children who tried more after failing had parents that encouraged them to be persistent. In contrast, although non-significant, children’s successful attempts were negatively correlated with all four parenting behaviors, suggesting that in general, children who had fewer successful attempts had parents who used more spatial language, praise, persistence-focused language, and scaffolding (see Table 1).

**Table 1:** Correlation between variables.

	Parent scaffolding	Parent praise	Parent persistence-focused language	Parent spatial utterances	Child successful insertion attempts	Child insertion attempts after failure	Child age	Difficulty score
Parent scaffolding	1							
Parent praise	0.256	1						
Parent persistence-focused language	0.174	0.325	1					
Parent spatial utterances	0.281	0.516^**^	0.100	1				
Child successful insertion attempts	−0.256	0.166	−0.092	−0.292	1			
Child insertion attempts after failure	0.049	0.185	0.463^**^	0.152	0.246	1		
Child age	−0.376^*^	0.188	−0.272	−0.088	0.282	0.016	1	
Difficulty score	0.042	0.040	0.131	0.106	−0.320	0.085	0.147	1

* *Correlation is significant at the 0.05 level (two tailed)*.

** *Correlation is significant at the 0.01 level (two tailed)*.

### Gender Differences

Finally to evaluate any gender differences, we ran a series of *t*-tests comparing boys to girls on each of our measured variables. There were no gender differences in terms of age (females *M*=45.2, *SD*=1.7; males *M*=44.39, *SD*=1.49) or difficulty of the puzzles (females *M*=6.89, *SD*=2.11; males *M*=6.29, *SD*=2.24). We did find a significant difference in the number of children’s attempts after failure, *t*(29)=2.19, *p*=0.021, 95% CI[−1.16, −0.04], with girls (*M*=1.64 attempts per minute, *SD*=0.99) attempting to place puzzle pieces more often after failure than boys (*M*=1.04 attempts per minute, *SD*=0.51). Thus, girls appeared to be more persistent than boys in their puzzle play. Further, we found that the difference in the amount of parents’ persistence-focused language directed to boys and girls approached significance *t*(29)=1.04, *p*=0.066, 95% CI[−0.15, 0.50], with parents using more persistence-focused utterances with girls (*M*=0.13, *SD*=0.16) than with boys (*M*=0.07, *SD*=0.12). None of the other parent or child variables differed as a function of child gender.

## Discussion

A large body of research has reported a positive relation between constructive play with toys like puzzles and developing spatial skills in children (e.g., Casey et al., 2008; Levine et al., 2012; Jirout and Newcombe, 2015; Bower et al., 2020). However, most of these studies were somewhat constrained, involving constructive play in a lab, and/or with a preselected and uniform set of constructive toys. Although the COVID-19 pandemic has kept many researchers away from the lab, it has offered us the opportunity to develop strategies for studying some of our basic research questions from a distance, by using tools like Zoom to examine what parents and children do in their own homes. Here, for the first time, we recorded parents and children interacting with puzzles of their choice at home and provided a descriptive account not only of their behaviors, but also of their behaviors in relation to the puzzles with which they most typically interact. Importantly, because we used Zoom, we may have observed more naturalistic behaviors than if we had been present in the home with a video recorder and an experimenter in the room. The experimenter kept her camera off, and thus parents and children may have forgotten her presence.

The most noteworthy finding from this descriptive study is the enormous variability we observed in both children and parents’ behaviors, and in the puzzles they selected for play. This study is the first of its kind in provide detailed characterization of the kinds of puzzles children have at their homes as well as the variability in parents’ and children’s behavior while engaging in home puzzle play. The puzzles themselves varied on a number of dimensions that we coded for difficulty. Some of the puzzles were typical jigsaw puzzles with interlocking pieces, while others were puzzle boards that had pieces with shapes that fit into specific places on a tray. Some of the puzzles had oversized pieces, presumably making them easier to place, while others even had a colorful background that matched the background of the puzzle pieces themselves, making it possible for children to use perceptual cues like color to match the pieces to their correct location. Some children played with puzzles that had less than 10 pieces, while other children played with 40 or 50 piece puzzles. No two play sessions were quite alike. These differences in the puzzles that children actually play with every day provide a context for studies of children’s puzzle play that have used a narrow set of puzzles. Researchers often assume that findings from the lab uncover processes involved in children’s puzzle play that reflect developmental changes in spatial ability. However, the variability in the types of puzzles available in children’s homes has raised the possibility that participants in lab studies might differ substantially in their familiarity with the experimental stimuli.

Besides variability in the puzzles, there was also a great deal of variability in both parents’ and children’s behavior when interacting with the puzzles. There were a large number of parents who engaged in very few scaffolding behaviors, and very little spatial language, praise, and persistence-focused language during the parent-child interactions. Most parents fell somewhere in the middle of the range, but there were also parents that produced an incredibly large amount of these behaviors, some with over 60 scaffolding behaviors in a 10-min play session, and upward of 30–40 praise and spatial language utterances. Further, parents who tended to use more spatial language also tended to use more praise and persistence-focused language, as evidenced by the significant correlations between these variables.

Children’s behavior also varied widely, with some of our participants attempting to place pieces into the puzzles less than 10–20 times, alongside almost a third of our sample producing more than 50 attempts. Their accuracy varied just as widely: Most of the children placed less than 20 pieces correctly in the 10-min session, but some placed more than 30. Older children tended to place more pieces correctly than younger children.

Given this large amount of variability and our small sample size, it is unsurprising that we found few significant correlations between our variables. However, our results do suggest some basic patterns. Specifically, there were few relations with child age in our data, likely reflecting, in part, the relatively narrow age range we sampled. More surprising, despite the wide variation in puzzle difficulty, there was little relation between the level of puzzle difficulty and child age, child behavior, or parent behavior. Parents also showed some evidence of being sensitive to children’s need for help. More persistence-focused language was related to more child attempts after failure. Interestingly, there was a hint that children’s successful attempts were negatively correlated with all four parenting behaviors. If confirmed in a larger sample, this pattern would suggest that parents’ language and scaffolding are related to children’s success in puzzle play. Specifically, it is possible that parents recognized when children were having a difficult time and used more language and scaffolding to direct them. Likewise, it is also possible that parents’ behavior impacted their children’s behavior. Indeed, children who attempted to place more puzzle pieces after failure also tended to have parents who encouraged them more, thus it is possible that parents’ persistence-focused language drove children to try harder.

Altogether, the variability we found in the puzzles themselves and in parent-child behaviors suggests that lab-based studies that impose a large number of constraints on children’s behavior might not fully represent how children interact with spatial toys in their everyday environments. It is especially noteworthy that our sample was not particularly diverse. Indeed, most of our families were middle to high income, and even then, we had to eliminate eight families because they did not have two puzzles in their homes. While our sample was not ethnically and economically diverse and this limitation hinders our confidence to generalize our findings to a wider population, we expect that in a more diverse sample, we are likely to see considerably more variability than reported here. Lower income families, for example, might not have as many puzzles at home as middle to higher income families, and as a result, children’s behavior when engaging in spatial play might differ systematically by SES. Further, the puzzles we observed here, while variable, were all characteristic of toys in Western, industrialized countries. It is likely that the types of spatial toys available cross-culturally vary significantly, which could, in-turn, affect the types of spatial play in which children engage.

This is not to suggest that lab-based studies are not useful or important; indeed, they have provided the basis for even the current investigation. Indeed, imposing constraints on children’s behavior allow us to narrow the focus of our research questions and ask more about the causal relations between variables. Further, it is important to acknowledge that the observational nature of this study was also limited in that the presence of the researcher, even with the camera off, may have changed parents’ behavior in a way that is systematically different from completely naturalistic behavior. Nevertheless, this work highlights the enormous amount of variability that exists in children’s spatial play at home in a very narrow sample, which has important implications for the conclusions we draw about lab-based studies that impose even more constraints on children’s behavior.

It is also important to note that despite the large amount of variability reported here, there are some relationships documented in previous literature that were also evident in the current sample, speaking to their robustness. For example, similar to our results, several studies have shown that parents provide more assistance to younger versus older children during puzzle-building tasks (Wertsch et al., 1980; Casasola et al., 2017), suggesting that parents might adjust their behavior to fit different children’s needs. Finally, we found several gender differences suggesting that girls were more persistent than boys, making more attempts to place pieces into the puzzle after failure, and that parents used more persistence-focused language with girls than with boys and gave girls more difficult puzzles. Gender differences in children’s spatial ability and spatial play have also been reported in previous literature, usually attributing more advanced spatial skills to boys than girls, but these findings are controversial (Baenninger and Newcombe, 1989; Levine et al., 2012) and require further research.

## Conclusion

In conclusion, despite its descriptive and non-causal nature, the current study informs us about the types of variability in spatial toys and spatial play we might expect in real-world settings and can help us contextualize the conclusions we draw from lab-based studies. Given the wide variability of puzzles available in children’s homes, future research could examine how the different characteristics of puzzles determine the nature of the parent-child interactions and what aspects of these interactions support spatial skills development. Our study also suggests that more large-scale, naturalistic studies of children’s spatial play in the home could be incredibly informative, providing us with important information about what types of spatial toys best promote the development of spatial skills, and how the types of toys interact with both child and parent characteristics over time.

## Data Availability Statement

The datasets and coding manuals presented in this study can be found in online repositories. The names of the repository/repositories and accession number(s) can be found at: LoBue, V., Pochinki, N., Oakes, L., and Casasola, M. (2021). Natural variability in parent-child puzzle play at home. *Databrary* available at: https://nyu.databrary.org/volume/1334 (Accessed June 29, 2021).

## Ethics Statement

The studies involving human participants were reviewed and approved by the Rutgers University Institutional Review Board. Written informed consent to participate in this study was provided by the participants’ legal guardian/next of kin.

## Author Contributions

NP, VL, LO, and MC drafted the manuscript. NP collected the data. NP and DR oversaw coding. All authors designed the study and approved the final version of the manuscript.

## Funding

This research and preparation of this manuscript were supported by a grant from the National Science Foundation (DS 1823489) to MC, LO, and VL. The funding agencies had no role in the design of the study or the collection, analysis, and interpretation of data or in writing the manuscript, apart from their financial contribution.

## Conflict of Interest

The authors declare that the research was conducted in the absence of any commercial or financial relationships that could be construed as a potential conflict of interest.

## Publisher’s Note

All claims expressed in this article are solely those of the authors and do not necessarily represent those of their affiliated organizations, or those of the publisher, the editors and the reviewers. Any product that may be evaluated in this article, or claim that may be made by its manufacturer, is not guaranteed or endorsed by the publisher.

## Footnotes

^1^ www.datavyu.org
